# Extracellular Vesicles Enriched in hsa-miR-301a-3p and hsa-miR-1293 Dynamics in Clear Cell Renal Cell Carcinoma Patients: Potential Biomarkers of Metastatic Disease

**DOI:** 10.3390/cancers12061450

**Published:** 2020-06-02

**Authors:** Francisca Dias, Ana Luísa Teixeira, Inês Nogueira, Mariana Morais, Joana Maia, Cristian Bodo, Marta Ferreira, Alexandra Silva, Manuela Vilhena, João Lobo, José Pedro Sequeira, Joaquina Maurício, Jorge Oliveira, Klaas Kok, Bruno Costa-Silva, Rui Medeiros

**Affiliations:** 1 Molecular Oncology and Viral Pathology Group, IPO-Porto Research Center (CI-IPOP), Portuguese Oncology Institute of Porto (IPO-Porto), Research Center- LAB2, E Bdg 1st floor, Rua Dr António Bernardino de Almeida, 4200-072 Porto, Portugal; Francisca.Carvalho.Dias@ipoporto.min-saude.pt (F.D.); inescmnogueira@gmail.com (I.N.); mariana.gomes.morais@ipoporto.min-saude.pt (M.M.); ruimedei@ipoporto.min-saude.pt (R.M.); 2 Institute of Biomedical Sciences Abel Salazar, University of Porto (ICBAS-UP), Rua Jorge Viterbo Ferreira 228, 4050-513 Porto, Portugal; 3 Research Department of the Portuguese League Against Cancer Regional Nucleus of the North (LPCC-NRN), Estrada da Circunvalação 6657, 4200-177 Porto, Portugal; 4 Systems Oncology Group, Champalimaud Research, Champalimaud Centre for the Unknown, Av. Brasília, 1400-038 Lisbon, Portugal; joana.maia@research.fchampalimaud.org (J.M.); cristian.bodo@research.fchampalimaud.org (C.B.); bruno.costadasilva@research.fchampalimaud.org (B.C.-S.); 5 Graduate Program in Areas of Basic and Applied Biology (GABBA), University of Porto, 4200-135 Porto, Portugal; 6 Department of Medical Oncology, Portuguese Oncology Institute of Porto (IPO-Porto), Rua Dr António Bernardino de Almeida, 4200-072 Porto, Portugal; marta.ribeiro.ferreira@ipoporto.min-saude.pt (M.F.); jmauricio@ipoporto.min-saude.pt (J.M.); 7 Department of Urology, Portuguese Oncology Institute of Porto (IPO-Porto), Rua Dr António Bernardino de Almeida, 4200-072 Porto, Portugal; alexandrasilva@ipoporto.min-saude.pt (A.S.); lucindavieira@ipoporto.min-saude.pt (M.V.); jorge.oliveira@ipoporto.min-saude.pt (J.O.); 8 Department of Pathology, Portuguese Oncology Institute of Porto (IPO-Porto), Rua Dr António Bernardino de Almeida, 4200-072 Porto, Portugal; jpedro.lobo@ipoporto.min-saude.pt; 9 Cancer Biology and Epigenetics Group, IPO-Porto Research Center (CI-IPOP), Portuguese Oncology Institute of Porto (IPO-Porto), Research Center- LAB3, F Bdg 1st floor, Rua Dr António Bernardino de Almeida, 4200-072 Porto, Portugal; jose.leite.sequeira@ipoporto.min-saude.pt; 10 Department of Genetics, University Medical Center Groningen (UMCG), University of Groningen, Hanzeplein 1, 9713 GZ Groningen, P.O. Box 30.001, 9700 RB Groningen, The Netherlands; k.kok@umcg.nl; 11 Faculty of Medicine, University of Porto (FMUP), Alameda Prof. Hernâni Monteiro, 4200-319 Porto, Portugal; 12 Biomedical Research Center (CEBIMED), Faculty of Health Sciences of Fernando Pessoa University (UFP), Praça 9 de Abril 349, 4249-004 Porto, Portugal

**Keywords:** clear cell renal cell carcinoma, extracellular vesicles, microRNAs, biomarkers

## Abstract

Clear cell renal cell carcinoma (ccRCC) is the most aggressive subtype of kidney cancer and up to 40% of patients submitted to surgery with a curative intent will relapse. Thus, the aim of this study was to analyze the applicability of an Extracellular vesicle (EV) derived miRNA profile as potential prognosis biomarkers in ccRCC patients. We analyzed a nine-miRNA profile in plasma EVs from 32 ccRCC patients with localized disease (before and after surgery) and in 37 patients with metastatic disease. We observed that the levels of EV-derived hsa-miR-25-3p, hsa-miR-126-5p, hsa-miR-200c-3p, and hsa-miR-301a-3p decreased after surgery, whereas hsa-miR-1293 EV-levels increased. Furthermore, metastatic patients presented higher levels of hsa-miR-301a-3p and lower levels of hsa-miR-1293 when compared to patients with localized disease after surgery. Functional enrichment analysis of the targets of the four miRNAs that decreased after surgery resulted in an enrichment of terms related to cell cycle, proliferation, and metabolism, suggesting that EV-miRNA enrichment in the presence of the tumor could represent an epigenetic mechanism to sustain tumor development. Taken together, these results suggest that EVs content varies depending on the presence or absence of the disease and that an increase of EV-derived hsa-miR-301a-3p, and decrease of EV-derived hsa-miR-1293, may be potential biomarkers of metastatic ccRCC.

## 1. Introduction

Renal cell carcinoma (RCC) is the most common solid cancer of the adult kidney and one of the most lethal urologic malignancies [1,2,3]. The most common histological subtype is clear cell RCC (ccRCC), which arises from the proximal tubular epithelial cells of the nephron and accounts for approximately 80% of all RCC cases [4]. CcRCC development is associated with two key cancer hallmarks: induction of angiogenesis and metabolic reprogramming, with several studies implying that ccRCC can be considered a metabolic disease [5,6,7,8]. In fact, the majority of known ccRCC related genes interact with cell metabolism pathways and are involved in energy, nutrient, iron and oxygen sensing [9]. One of the most well characterized pathways involved in the development of ccRCC, and the most studied as well, is the VHL/HIF pathway [10,11]. The loss or inactivation of pVHL function in ccRCC leads to a state of “pseudohypoxia” where stabilized HIF-1α and HIF-2α induce the transcription of hypoxia responsive genes, resulting in alterations such as increased glucose and ribose metabolism, pH deregulation, cell proliferation, and angiogenesis, giving ccRCC a high metastatic potential [1,9,12,13,14]. 

Actually one third of ccRCC patients present metastatic disease at the time of diagnosis, and up to 40% of patients submitted to surgery with a curative intent, will relapse within a five-year period [15]. The therapeutic options for ccRCC patients are limited since the hypoxic microenvironment makes these tumors chemo- and radio- resistant, leaving targeted therapies and immunotherapies as the only options available. However, patients submitted to these therapies tend to develop resistance to therapy within a short period of time [16,17]. Despite the fact that metastatic ccRCC remains incurable, the prognosis for recurrent ccRCC is variable and the detection of early relapse could have an impact on patients’ prognosis [18]. Thus, there is an urgent need for the definition of reliable biomarkers that can help stratify patients according to their metastatic risk. In the recent years, it became clear that one of the key players of tumor microenvironment modulation are the extracellular vesicles (EVs). EVs consist of a mixed population of microvesicles with different sizes, shed by cells, that enable cell-to-cell communication through the transport of active biomolecules from one cell to another [19,20]. Cancers have been found to highjack EV-mediated communication to facilitate several features of the multi-step metastatic process, including cell proliferation, immune suppression, epithelial-to-mesenchymal transition, migration, invasion, angiogenesis, and metastasis [5,21,22]. The release of EVs can be induced by several factors including, hypoxia, pH alterations, injury, platelet activation, irradiation and cellular stress, some of them involved in ccRCC progression [23]. One of the most studied classes of biomolecules carried by EVs are the microRNAs (miRNAs), which consist of small non-coding RNAs that are able to regulate gene expression at a post-transcriptional level, resulting in the attenuated translation of target mRNAs [24,25]. Thousands of protein-coding genes are regulated by miRNAs, and miRNAs are master regulators of diverse biological systems and have an impact in the body physiological responses [26,27]. Several EV-derived miRNAs have been studied and proposed as potential biomarkers in ccRCC [28,29,30,31,32,33,34,35,36,37,38]. However, due to the novelty of the field, most of the studies have been performed in cell lines and only a few used samples from ccRCC patients.

Therefore, the aim of this study is to analyse the impact of EV-derived miRNA profiles of 9 miRNAs (hsa-miR-25-3p, hsa-miR126-5p, hsa-miR-200c-3p, hsa-miR-210-3p, hsa-miR-301a-3p, hsa-miR-519d-3p, has-miR-1233-5p, hsa-miR-1246, and hsa-miR-1293) related to hypoxia and metabolism regulation in plasma EVs from ccRCC patients with localized disease and also in patients with metastatic disease.

## 2. Results

### 2.1. EVs Characterization

**Figure 1:**
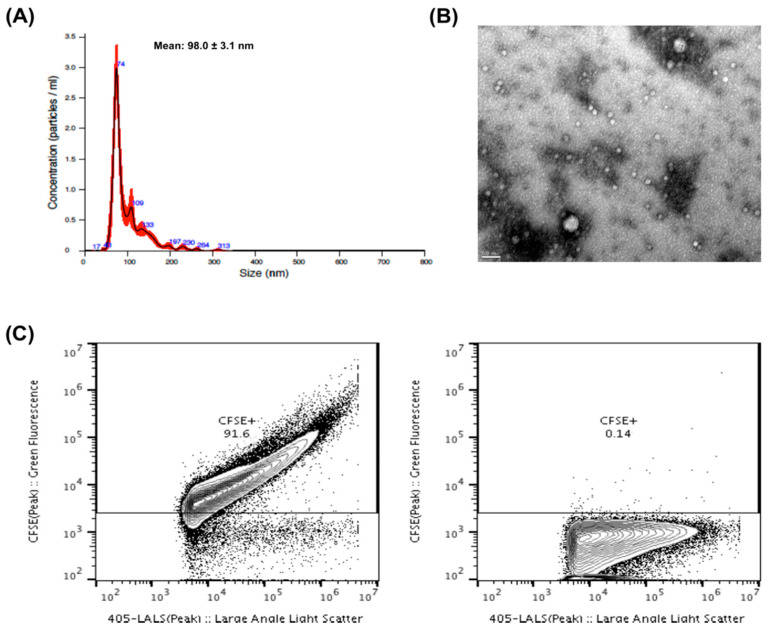
(**A**) Nanoparticle tracking analysis (NTA) of EVs derived from plasma of ccRCC patients. The red error bars indicate ± 1 standard error of the mean. (**B**) Transmission electron microscopy (TEM) of EVs from purified platelet-free plasma (PFP). The TEM image was acquired in the Histology and Electron Microscopy platform from I3S Porto using a Transmission Electron Microscope Jeol JEM 1400. (**C**) Proportion of fluorescent EVs as observed by flow cytometry from a sample EV isolate derived from a patient, either previously stained with CFSE (left panel) or without previous staining (right panel).

The EVs were characterized according to size, shape and purity. The NTA analysis indicated that the vast majority of isolated EVs presented a size range between 50 and 200 nm, which is consistent with the size of exosomes and small microvesicles (Figure 1A). A transmission electron microscopy (TEM) image (Figure 1B) shows the variability of sizes and morphology present in EVs from purified PFP. We also utilized EVs flow cytometry to confirm the purity of our EV isolates [39]. In all cases, more than 80% of particles present in our isolates corresponded to CFSE^+^ vesicular structures (Figure 1C).

### 2.2. EV-Derived miRNA Levels in ccRCC Patients’ Plasma Samples

Two patients from Group A relapsed during the follow-up and were removed from the analysis. The remaining 30 patients from group A are currently alive with no evidence of disease. In addition to that, two EV-derived miRNAs were excluded from the analysis. Hsa-miR-519d-3p was only detected in a small number of samples and hsa-1233-5p was not detected at all, which did not allow a statistical analysis. 

**Figure 2:**
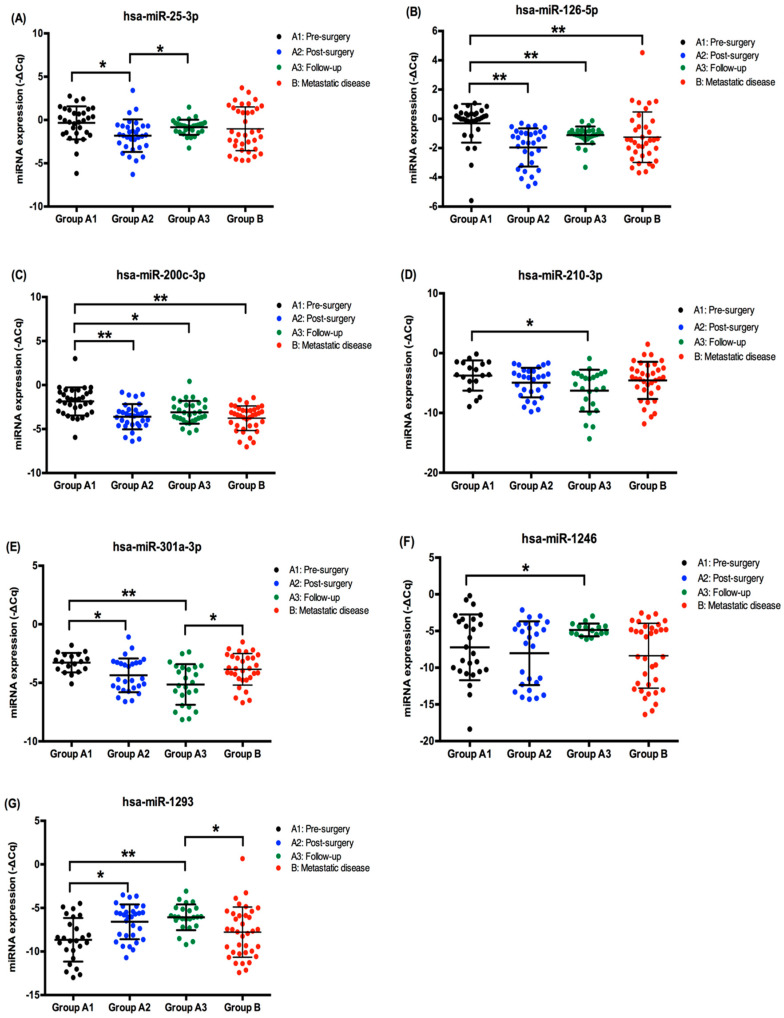
EV-derived miRNA expression (-ΔCq) in pre-surgery, post-surgery, and follow-up samples (Group A) and metastatic disease samples (Group B) of ccRCC patients. (**A**) hsa-miR-25-3p plasmatic EV-expression; (**B**) hsa-miR-126-5p plasmatic EV-expression; (**C**) hsa-miR-200c-3p plasmatic EV-expression; (**D**) hsa-miR-210-3p plasmatic EV-expression; (**E**) hsa-miR-301a-3p plasmatic EV-expression; (**F**) hsa-miR-1246 plasmatic EV-expression; (**G**) hsa-miR-1293 plasmatic EV-expression; * *p* < 0.05, ** *p* < 0.001.

The expression levels of the remaining eight miRNAs are represented in Figure 2. Focusing on the patients from Group A, we observed that the levels of EV-derived hsa-miR-25-3p (*p* = 0.003), hsa-miR-126-5p (*p* < 0.001), hsa-miR-200c-3p (*p* < 0.001) and hsa-miR-301a-3p (*p* = 0.006) decreased after surgery (Figure 2A–C,E). Hsa-miR-210-3p also decreased after tumor removal but the decrease was lower and only statistically significant when the localized disease samples were compared with the follow-up samples (*p* = 0.010) (Figure 2D). On the other hand, we observed that hsa-miR-1293 (*p* = 0.002) EV-levels increased after tumor removal (Figure 2G). Hsa-miR-1246 EV-levels also increased after tumor removal, but at a slower rate since the levels were only statistical significantly higher when we compared the localized and follow-up samples (*p* = 0.044) (Figure 2F). 

When we compare the patients from Group A with the patients from Group B, we observe two scenarios. First, we see that some miRNAs have differences in their expression levels when comparing the samples from localized disease (Group A) with the metastatic disease (Group B). This is the case for hsa-miR-126-5p and hsa-miR-200c-3p that are downregulated in the metastatic patients (*p* < 0.001 and *p* < 0.001, respectively), (Figure 2B,C). On the other hand, we observed differences in miRNA levels when we compare the follow-up samples with the metastatic samples, with the metastatic samples presenting higher levels of hsa-miR-301a-3p (*p* = 0.026) and lower levels of miR-1293 (*p* = 0.004) (Figure 2E,G). In addition to that, hsa-miR-210-3p presented a tendency for being upregulated (*p* = 0.053) and hsa-miR-1246 presented a tendency for being downregulated (*p* = 0.053), both in the metastatic samples, when compared with the follow-up group (Figure 2D,F).

Regarding the other clinical pathological characteristics, we observed that patients with localized disease with tumors larger than 7 cm presented higher levels of hsa-miR-126-5p (*p* = 0.013) (Table S1). Additionally, patients who smoke presented higher levels of hsa-miR-1293 (*p* = 0.006) and hsa-miR-210-3p (*p* = 0.034) when compared to non-smokers and ex-smokers, respectively. We did not observe any statistical differences between the EV-derived miRNAs and hypertension and *diabetes mellitus*.

### 2.3. EV-Derived miRNAs Impact on Overall Survival of Metastatic ccRCC Patients 

**Figure 3:**
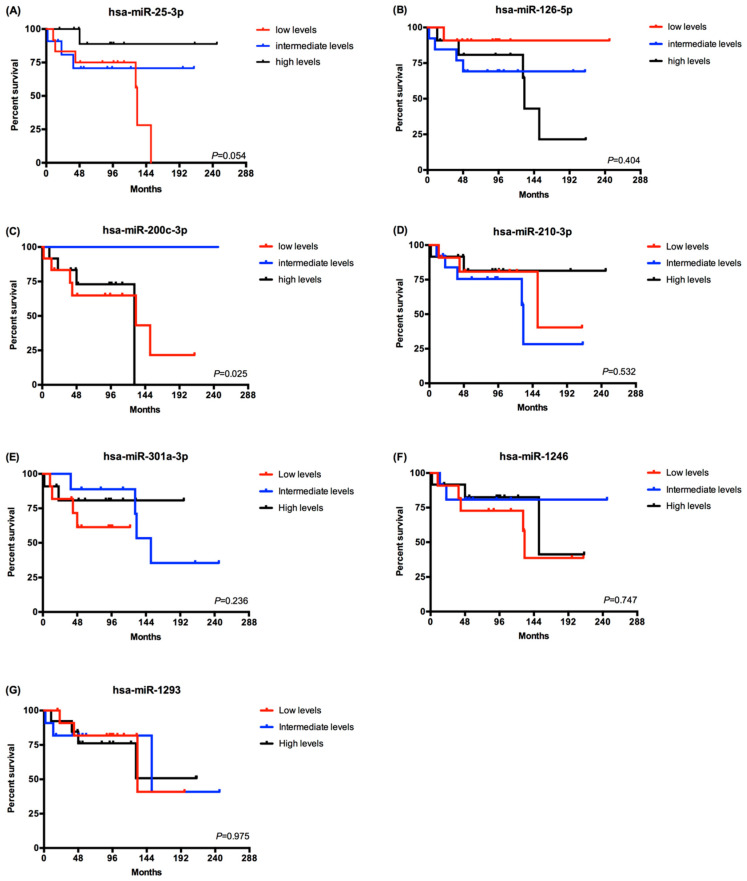
Overall survival analysis of metastatic ccRCC patients according to EV-derived miRNAs expression. Comparison of survival curves was made with Log-rank (Mantel-Cox) test. (**A**) hsa-miR-25-3p (low levels–12 patients; intermediate levels–11 patients and high levels–12 patients). (**B**) hsa-miR-125-5p (low levels–12 patients; intermediate levels–13 patients and high levels–11 patients). (**C**) hsa-miR-200-3p (low levels–12 patients; intermediate levels–12 patients and high levels–12 patients). (**D**) hsa-miR-210-3p (low levels–11 patients; intermediate levels–13 patients and high levels–12 patients). (**E**) hsa-miR-301a-3p (low levels–11 patients; intermediate levels–10 patients and high levels–11 patients). (**F**) hsa-miR-1246 (low levels–11 patients; intermediate levels–11 patients and high levels–12 patients). (**G**) hsa-miR-1293 (low levels–12 patients; intermediate levels–11 patients and high levels–12 patients).

Since the patients from group A were alive with no evidence of disease on their last observation, we carried out the survival analysis on the patients from group B (Figure 3). The patients were divided in tertiles according the EV-derived miRNA levels using the –ΔCq values of each miRNA (high, intermediate and low levels). Regarding the 8 EV-derived miRNAs analyzed, only the hsa-miR-200c-3p presented statistical significant differences. Patients with a high or low expression level had a lower overall survival compared to patients with intermediate levels of hsa-miR-200c-3p (log rank Mantel Cox test, *p* = 0.025) (Figure 3C). In addition to that, patients with lower levels of has-miR-25-3p presented a tendency for a lower overall survival (log rank Mantel Cox test, *p* = 0.054) (Figure 3A).

### 2.4. Hsa-miR-25-3p, hsa-miR-126-5p, hsa-miR-200c-3p, hsa-miR-301a-3p Overlaping Target Genes

**Figure 4:**
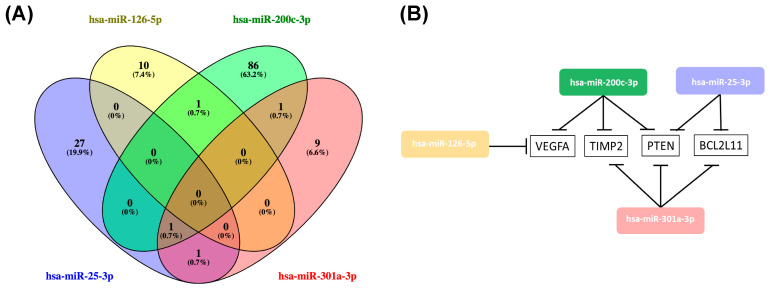
(**A**) Venn diagram of the validated target genes of hsa-miR-25-3p, hsa-miR-126-5p, hsa-miR-200c-3p and hsa-miR-301a-3p obtained using Venny 2.1 (https://bioinfogp.cnb.csic.es/tools/venny/). (**B**) Representation of the overlaping target genes of hsa-miR-25-3p, hsa-miR-126-5p, hsa-miR-200c-3p and hsa-miR-301a-3p.

Hsa-miR-25-3p, hsa-miR-126-5p, hsa-miR-200c-3p, and hsa-miR-301a-3p significantly decreased after tumor removal, suggesting that their expression may be related to the presence of the tumor. As such, we used miRTarBase (version 8.0), the largest known online database of validated miRNA:mRNA target interactions, to establish a network of the target mRNAs of this miRNA profile and further evaluate its impact on ccRCC [40]. According to miRTarBase there are a total of 1381 validated target genes for these four miRNAs, but we only focused on the the 135 whose miRNA:mRNA interaction was validated according to strong validation methods (such as luciferase reporter assay, western blot and qPCR) (Table S2). From the 135 validated target genes, *PTEN*, *VEGFA*, *TIMP2*, and *BCL2L11* were common to more than one of the miRNAs (Figure 3B). *PTEN* was the most common target, being regulated by hsa-miR-25-3p, hsa-miR-200c-3p, and hsa-miR-126-5p (Figure 4).

### 2.5. Functional Annotation and Pathway Enrichment Analysis of has-miR-25-3p, hsa-miR-126-5p, hsa-miR-200c-3p and hsa-miR-301a-3p Targets Network

**Figure 5:**
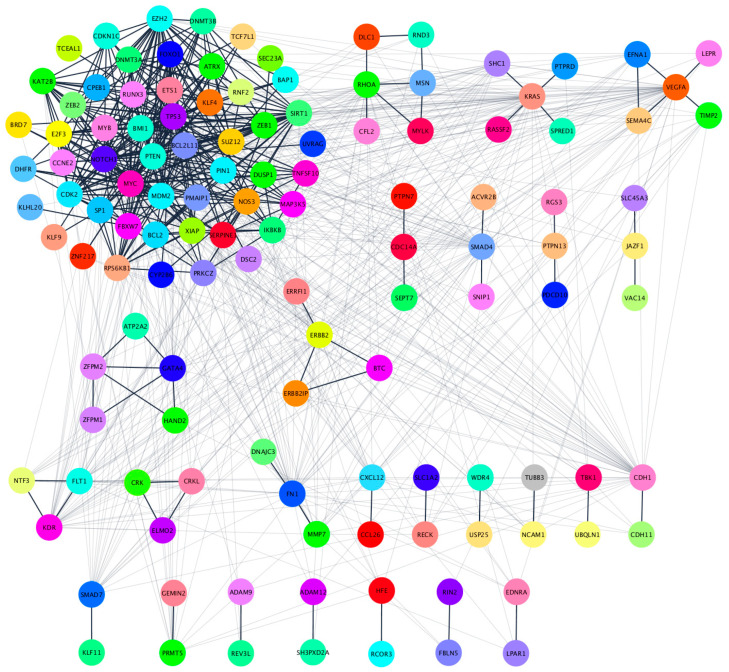
Clustered PPI network of the 134 target genes. Clustering was performed using the Markov clustering (MCL) algorithm in the clusterMaker2 Cytoscape app using an inflation value of 4.0. Proteins without any interaction partners within the network (singletons) are omitted from the visualization.

In order to explore the biological impact of the four-miRNA signature in patients with localized disease, we analyzed their 135 target genes with the STRINGapp from Cytoscape software (v3.7.2). A total of 134 of the 135 protein coding genes were filtered into a protein-protein interaction (PPI) network with 134 nodes and 889 edges that presented significant enrichment (*p* = 1.0 × 10^−16^). For a deeper understanding of the protein interactions we performed a Markov clustering (MCL), which resulted in the clustering of the proteins into 26 clusters according to their STRING interaction score (Figure 5, Table S3).

**Figure 6:**
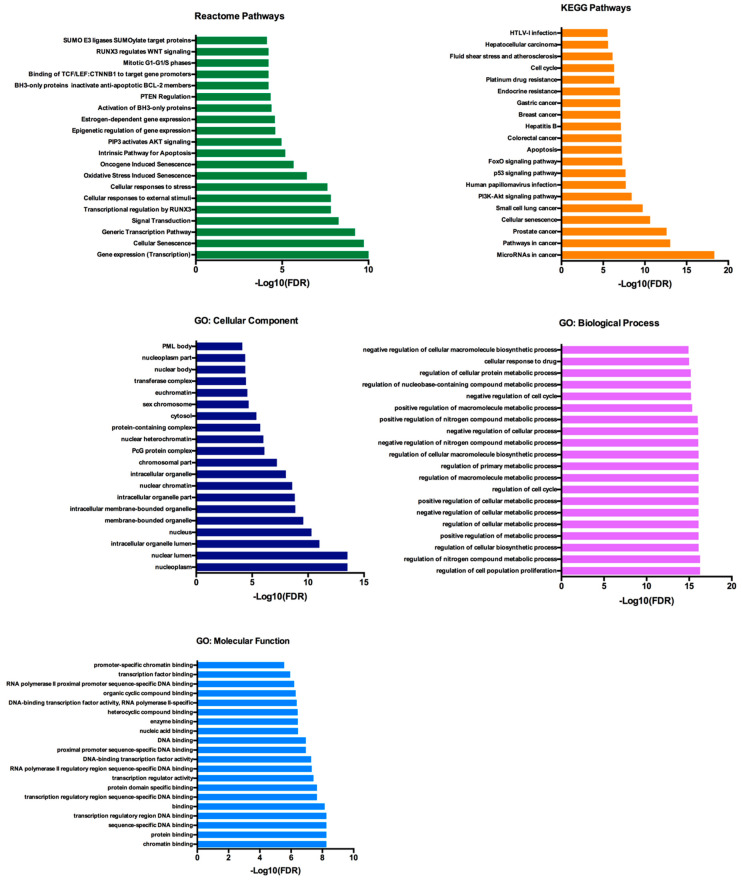
Reactome, KEGG and GO analysis of the 53 selected genes. The functional enrichment anaylis was made with the STRINGapp from Cytoscape.

For the functional enrichment analysis, we focused on the largest cluster, which reduced the initial network of 134 proteins to 53 proteins. This cluster contained 53 nodes, with PTEN and BCL2L11 among them, and 328 edges and retained the significant PPI enrichment (*p* = 1.0 × 10^−16^). The functional enrichment analysis was performed with an FDR threshold of 1%, and the redundant terms were eliminated using a redundancy cutoff of 0.5, which resulted in a total of 549 functional enriched terms among the Reactome, KEGG, and GO categories (Tables S4–S8). The top 20 enriched terms for each category are represented in Figure 6.

Among the functionally enriched terms in the Reactome and KEGG pathways we can find AKT, BCL-2, PTEN and p53 signaling. Regarding the GO terms, we can observe that terms related with the Biological Processes were mostly about cellular metabolic processes and also regulation of cell cycle and cell proliferation.

## 3. Discussion

It is now well established that the formation and maintenance of a cancer niche deeply relies in the surrounding microenvironment that is composed by different cell types such as endothelial cells, stem cells, fibroblasts and immune cells [41]. Moreover, EVs play an important role in the complex network of communication that occurs inside the tumor microenvironment and also between the tumor microenvironment and the rest of the human body. This is due to the bioactive molecules they transfer between cells that are able to make a biological impact through the alteration of the recipient cell phenotype.

In our study, we observed that patients with localized ccRCC presented an alteration of the pattern of plasmatic EV-derived miRNAs after tumor removal, with a decrease of hsa-miR-25-3p, hsa-miR-126-5p, hsa-miR-200c-3p, and hsa-miR-301a-3p, and an increase of hsa-miR-1293 approximately four months after surgery, suggesting that the presence of the tumor has a key role in the EV network that is established in the patients’ body. In fact, hsa-miR-25-3p, hsa-miR-126-5p, hsa-miR-200c-3p, and hsa-miR-301a-3p have already been reported in EVs, with an impact on cell proliferation, pre-metastatic niche formation, invasion, and metastization in other tumor models [42,43,44,45]. When we looked into the validated target genes of the four miRNAs that decreased after tumor removal, we observed that *PTEN* was the most common target, being regulated by hsa-miR-25-3p, hsa-miR-200c-3p and miR-301a-3p. Since one miRNA is capable of regulating several targets, and the same mRNA can be regulated by multiple miRNAs, some authors defend that the repressing capability of a single miRNA is quite modest and not capable of inducing significant changes on protein expression [46]. However, the fact that *PTEN* is repressed by three of the studied miRNAs suggests that this gene might be under a stronger repressing environment in the patients with localized disease. PTEN is a tumor suppressor gene whose major function relies on the inhibition of the PI3K/Akt pathway and consequent regulation of pathways related to cell growth and proliferation [47,48]. This gene is frequently altered in ccRCC and recent data from The Cancer Genome Atlas (TCGA) showed that *PTEN* loss of function in ccRCC patients was associated with a more vigorous cell metabolism and cell growth and also with a worse prognosis in survival and disease recurrence [49,50]. Moreover, a study performed by Zhang and colleagues demonstrated that astrocyte-derived EVs transferred *PTEN*-targeting miRNAs to metastatic cells, with an impact on mRNA and protein downregulation [51]. This epigenetic silencing of *PTEN* in metastatic cells leads to CCL2 secretion and myeloid cell recruitment, which promoted metastatic cell expansion through reduced apoptosis and enhanced proliferation through PI3K/Akt pathway activation [51]. 

Our functional annotation analysis on the validated target genes of hsa-miR-25-3p, hsa-miR-126-5p, hsa-miR-200c-3p and hsa-miR-301a-3p showed that the most enriched terms were related to cell growth, proliferation and metabolism, with *PTEN*, *AKT*, *BCL-2* and *p53* signalling among the most enriched terms. Since these EV-derived miRNAs decrease after tumor removal, our hypothesis is that their EV-enrichment in the presence of the tumor could represent an epigenetic silencing mechanism used by ccRCC to sustain tumor development and growth through activation of the PI3K/Akt pathway. In fact, despite the fact that the overall mutation rate of PI3K/Akt pathway in ccRCC is relatively low, the overall activation of PI3K/Akt in ccRCC is high compared to other cancers, suggesting that the dyregulation of the PI3K/Akt pathway in ccRCC could be a consequence of epigenetic mechanisms mediated by EVs [52,53,54]. However, additional studies are necessary in order to validate this hypothesis.

Regarding the metastatic patients, EV-derived hsa-miR-301a-3p and hsa-miR-1293 were the most promising miRNAs to differentiate patients in follow-up with no evidence of disease from patients with metastatic disease. Hsa-mir-301a-3p presented a decreasing expression pattern after tumor removal and its levels kept decreasing until follow-up. However, the expression levels were significantly increased in the metastatic group, suggesting that this miRNA could play an important role in the metastization process and might have the potential to be used as prognostic biomarker. To support this hypothesis, a study performed by Wang and colleagues showed that, under a hypoxic microenvironment, pancreatic cancer cells generate EVs enriched in hsa-miR-301a-3p that induce M2 macrophages polarization through the PTEN/PI3K/Akt signalling pathway and promote metastization [43]. In addition to that, Yan and colleagues described an oncogenic role for hsa-miR-301a-3p in Laryngeal Squamous Cell Carcinoma development through inhibition of Smad4 and participation in the EMT process [55]. Regarding hsa-miR-1293, we observed the opposite effect since its EV-derived levels started to gradually increase after tumor removal until follow-up, but were significantly decreased in the metastatic group, suggesting a tumor suppresser role for this miRNA. In fact, Takagawa reported that hsa-miR-1293 was able to suppress in vivo tumor growth in a xenograft mouse model through the inhibition of BRD4 and DNA repair genes, and hypothesized that this miRNA could be a candidate for the development of miRNA-based therapeutics [56]. In addition to that, TIMP-1 is also a validated target of hsa-miR-1293 and its increased expression is associated with a worse prognosis in ccRCC patients, which reinforces the tumor suppressor role of hsa-miR-1293. [57,58]. 

Regarding the impact of the EV-derived miRNA profile in the metastatic patients’ overall survival, only hsa-miR-200c-3p presented significant, but contradictory, results since both its high and low levels were associated with a worse survival. Although based on a limited number of cases, our results may highlight the importance of a balance in EV-derived miRNA abundance and how disruption of that balance can have an impact on the patients’ prognosis. To the best of our knowledge this is the first study monitoring the differences of EV-derived miRNAs in several time points during the course of a one-year follow-up period of ccRCC patients with localized disease. This type of study is useful for a better understanding of the disease impact on the host EV dynamics and search for potential new prognosis biomarkers, such as hsa-miR-301a-3p and hsa-miR-1293. However, the small samples size in our study may limit the ability to distinguish meaningful differences, especially in terms of the survival analysis in the metastatic group, being essential to the replication of the associations reported in a larger sample size. In addition to that, the patients included in this study should continue to be monitored in order to get a deeper understanding of EV-miRNA enrichment during the course of the disease. 

## 4. Materials and Methods 

### 4.1. Ethics Statment

This study was conducted according to the principles of the Helsinki Declaration, having been approved by the ethics committee of the Portuguese Oncology Institute of Porto (IPO-Porto) (project reference: 251/015). All individuals signed a written informed consent in order to participate in the study.

### 4.2. Study Population

The analysis of the EV-derived miRNA profile was conducted through a hospital-based study involving a total of 69 ccRCC patients. All individuals were Caucasian from the north of Portugal, with histopathological diagnosis of ccRCC, and admitted and treated at the IPO-Porto between November 2015 and June 2019. The patients were divided into two groups: 32 patients diagnosed with localized disease that underwent surgical intervention composed Group A; and Group B was composed of 37 patients with metastatic disease. Clinical characteristics of patients were obtained from their medical records (Table 1). Blood from Group A patients was collected three times during the study: before undergoing surgery, approximately 4 months after surgery, and approximately 1 year after surgery, while from Group B, patients’ blood was collected once. All blood collections were performed during the morning period and stored at 4 °C immediately until sample processing.

Tumor classification and staging was established according to the tumor-node-metastasis (TNM) classification system of the American Joint Committee on Cancer (AJCC) 8th edition (2018) and the International Society of Urological Pathology (ISUP) Classification of Renal Neoplasia [59].

### 4.3. EVs Isolation

EVs were isolated from the plasma fraction using the Total Exosome Isolation from Plasma Kit (Invtrogen^TM^, Waltham, MA, USA) with additional protocol optimizations. Firstly, 8 mL of peripheral blood was collected from the patients in EDTA tubes and centrifuged 5 min at 1800× *g* to obtain the plasma fraction. The plasma fraction was then centrifuged 3 additional times at increasing speeds (300× *g*, 2100× *g* and 10,000× *g*) for a period of 15 min each in order to obtain platelet-free-plasma (PFP). After centrifugation, the supernatant was recovered and filtered through a 0.22 μM filter (GE Healthcare Whatman^TM^, Chicago, IL, USA). After a 10-min treatment with proteinase K, the Total Exosome Isolation (TEI) reagent was added to 200 μL of PFP and the solution was incubated for 30 min at 4 °C. The precipitated EVs were recovered by 5 min centrifugation at 10,000× *g* at room temperature. The pellet containing the pre-enriched EVs was resuspended in filtered PBS (0.22 µm membrane filters) and stored at −80 °C until further analysis.

### 4.4. EVs NTA Analysis

Samples were analyzed for particle concentration and size distribution by the NS300 Nanoparticle Tracking Analysis (NTA) system (NanoSight–Malvern Panalytical, Malvern, UK). Samples were pre-diluted in filtered PBS to achieve a concentration within the range for optimal NTA analysis. Video acquisitions were performed using a camera level of 16 and a threshold between 5 and 7. Five to nine videos of 30 s were captured per sample. Analysis of particle concentration per mL and size distribution was performed with NTA software v3.4.

### 4.5. Quantification of Vesicular Structures by EVs Flow Cytometry

We employed EVs flow cytometry procedure for quantification of vesicular structures in our EV isolates, as recently described by Maia and colleagues [39]. Briefly, 2 × 10^9^ particles of purified EVs were mixed with 40 µL of PBS containing Carboxyfluorescein Diacetate Succinimidyl Ester (CFSE – Thermo Fisher Scientific-LTI C34554, Waltham, MA, USA) in a final concentration of 40 µM and incubated for 90 min at 37 °C. For removal of unbound CFSE, size exclusion chromatography (SEC) columns (iZON - qEV original columns SP1, Oxford, UK) were used. Samples containing unstained or stained EVs, and appropriate controls, were diluted up to 500 µL of PBS and processed by qEV following manufacturer’s instructions. EVs-enriched fractions #7, #8 and #9 were then compiled and retrieved for analysis with the Flow Cytometer Apogee A60-Micro-Plus (Apogee Flow Systems, London, UK). The A60-Micro-Plus machine is equipped with three spatially separated lasers (488 nm – Position C, 405 nm – Position A and 638 nm–Position B), 7 fluorescence color detectors (525/50, LWP590, 530/30, 574/26, 590/40, 695/40, 676/36), and 3 light scatter detectors (SALS, MALS and LALS). For internal control across assays, before each FC experiment, we used two mixes of beads (Apogee–1493 and Apogee-1517). Before being loaded, samples were diluted in filtered PBS (0.22 µm membrane filters) to bring their concentration within the operational range of the equipment (maximum of 3000 events/second). All samples were run at a flow rate of 1.5 µL/minute using a 405 nm LALS threshold of 70. The 405 nm LALS PMT noise level was monitored and always maintained below 0.35. For the experiments depicted, the stopping criteria utilized was the number of events acquired, so samples were run until a minimum of 250,000 events was reached. The acquired data were exported and analyzed with FlowJo software v10.4.2 (FlowJo LLC, Ashland, OR, USA).

### 4.6. miRNA Extraction and cDNA Synthesis

MiRNA isolation and purification of cell-derived EVs was done using the Plasma/Serum RNA Purification Mini Kit from NORGEN (Norgen Biotek Corporation, Therold, ON, Canada) according to the manufacturer supplementary protocol for EV RNA purification from EVs already isolated from precipitation methods. RNA concentration and purity were measured using the NanoDrop Lite spectrophotometer (Thermo Scientific^®^, Waltham, MA, USA) and served as template for cDNA synthesis using a TaqMan^TM^ Advanced miRNA cDNA Synthesis Kit (Applied Biosystems^®^, Foster City, CA, USA) according to the manufacturer protocol.

### 4.7. Quantitative Real Time PCR

MiRNA expression levels were analyzed by quantitative real-time PCR. The reactions were carried out in a StepOnePlus^TM^ qPCR Real-Time PCR machine, in a volume of 10 µL containing 1× TaqMan^TM^ Fast Advanced Master mix (Applied Biosystems), with 1X TaqMan^TM^ Advanced miRNA Assays probes (hsa-miR-25-3p 477994_mir; hsa-miR-126-5p 477888_mir; hsa-miR-200c-3p 478351_mir; hsa-miR210-3p 477970_mir; hsa-miR-301a-3p 477815_mir; hsa-miR-519d-3p 478986_mir; hsa-miR-1233-5p 479549_mir; hsa-miR-1246 477881_mir, and hsa-miR-1293 478692_mir-Applied Biosystems), and 2.5 µL cDNA. For miRNA expression normalization two housekeeping controls were used: hsa-let7a-5p (478575_mir-Applied Biosystems) and hsa-miR-16-5p (477860_mir-Applied Biosystems). These housekeeping miRNAs were chosen based on the fact that they are reported as typical EVs cargo [60]. The amplification conditions were as follows: holding stage 95 °C for 20 s, followed by 45 cycles of 95 °C for 1 s and 60 °C for 20 s. Three technical replicates were made for each sample. Data analysis was done using StepOne^TM^ Sofware v2.2 (Applied Biosystems) with the same baseline and threshold set for each plate, in order to generate quantification cycle (Cq) values for all the miRNAs in each sample.

### 4.8. Statistical Analysis

Statistical analyses were done using IBM SPSS Statistics software for Windows (Version 22.0). According the mRNAs levels distribution, the Student t´test or Mann–Whitney U test were used in order to evaluate any statistical differences in the normalized expression of the EV-derived miRNAs. The quality of the housekeeping miRNAs was tested using the BestKeeper software [61]. Only hsa-let-7a-5p presented a stable behavior among all samples and was used to data normalization. The Kaplen Meier method and Log Rank test were used to establish the association of the EV-derived mRNA levels (low, intermediate, and high) to the overall survival. 

### 4.9. Protein-Protein Interaction (PPI) Network and Cluster Analysis

The Search Tool for the Retrieval of Interacting Genes (STRING) database is an online tool that is used to develop protein-protein interaction (PPI) networks [62]. We used the STRINGapp in the Cytoscape software (v3.7.2) to construct the protein interaction relationship of the selected target genes encoding proteins. Those with a combined score of >0.4 were selected as significant. In order to get a better understanding of the resulting PPI network, we grouped the proteins according to the strength of their STRING interaction score using the clusterMaker app for Cytoscape to perform Markov clustering (MCL) [63,64]. 

### 4.10. Functional Annotation and Pathway Enrichment Analysis

Gene ontology analysis (GO) is a common useful method for annotating genes and gene products, allowing the identification of characteristic biological attributes for transcriptome data. The functional enrichment analysis of GO, Reactome and Kyoto Encyclopedia of Genes and Genomes (KEGG) pathways was performed with the enrichment anaylsis tool of the STRINGapp, with a False Discovery Rate (FDR) of *p* < 0.01 as significance threshold. The enrichment results were filtered in order to remove redundant terms. The redundancy filtering takes the list of enriched terms sorted by FDR value and removes the terms that are too similar to any of the previous, better scoring terms that were not themselves removed. The similarity between two terms is measured by the Jaccard índex of the sets of genes annotated by the two terms. A term is added to the filtered list only if it has Jaccard similarity less than the used specified redundancy cutoff to any other term already in the filtered list.

## 5. Conclusions

Taken together, these results suggest that EVs content varies depending on the presence or absence of the disease and an increase of EVs enriched in hsa-miR-301a-3p, and decrease of EVs enriched in hsa-miR-1293, may be potential biomarkers of metastatic disease in ccRCC patients.

## Figures and Tables

**Table 1:** Clinical-pathological characteristics of the study population. Group A is constituted by the patients with localized disease and Group B is constituted by the patients with metastatic disease.

Clinical-Pathological Characteristics		Group A (N = 32)		Group B (N = 37)	
		N	%	N	%
*Gender*					
	Male	24	75.0	26	70.3
	Female	8	25.0	11	29.7
*Age*					
	Average ± SD	61.9 ± 12.4		62.4 ± 9.9	
*Type of surgery*					
	Partial nephrectomy	18	56.3	0	0.0
	Radical nephrectomy	14	43.8	34	91.9
	No surgery	0	0	3	8.1
*Tumor size*					
	<7 cm	24	75.0	9	24.32
	≥7 cm	8	25.0	22	59.46
	Undetermined	0	0	6	16.22
*T*					
	T1	17	53.1	10	27.03
	T2	1	3.1	6	16.22
	T3	13	40.6	16	43.24
	T4	0	0	3	8.12
	Tx	1	3.1	2	5.40
*N*					
	N0	0	0	15	40.54
	N1–N2	0	0	5	13.51
	Nx	0	0	17	45.95
*M*					
	M0	27	100	22	59.46
	M1	0	0	15	40.54
	Mx	5	0	0	0.0
*Clinical Stage*					
	I	17	53.1	10	27.03
	II	1	3.1	6	16.22
	III	13	40.6	16	43.24
	IV	0	0	3	8.12
	Not available	1	3.1	3	5.40
*ISUP classification*					
	1	2	6.3	1	2.70
	2	18	56.3	14	37.84
	3	11	34.3	8	21.62
	4	1	3.1	9	24.32
	Not available	0	0	5	13.51
*Smoking status*					
	Smoker	6	18.8	3	8.11
	Ex-smoker	11	34.4	7	18.92
	Non-smoker	14	43.8	27	72,97
	Not available	1	3.1	0	0.0
*Hypertension*					
	Yes	20	62.5	15	40.54
	No	11	34.4	22	59.45
	Not available	1	3.1	0	0.0
*Diabetes mellitus*					
	DM I	0	0.0	0	0.0
	DM II	9	28.1	13	35.14
	No	22	68.8	23	62.16
	Not available	1	3.1	1	2.70

## Supplementary Materials

The following are available online at https://www.mdpi.com/2072-6694/12/6/1450/s1, Table S1: Association of EV-derived miRNA profile expression with Clinical-pathological characteristics of patients with localized disease (Group A)., Table S2: Hsa-miR-25-3p, hsa-miR-126-5p, hsa-miR-200c-3p and hsa-miR-301a-3p mRNA targets validated with strong evidence according miRTarBase V8.0, Table S3: Protein-protein-interaction (PPI) network clusters according to Marckov Clusterring (MCL) analysis, Table S4: Reactome pathway enrichment analysis for terms with FDR p value <0.01, Table S5: KEGG pathway enrichment analysis, Table S6: Gene Ontology (GO) Cellular Components enrichment analysis, Table S7: Gene Ontology (GO) Biological data enrichment analysis, Table S8: Gene Ontology (GO) Molecular Function data enrichment analysis.
