# Supplementary material for: Extracellular Vesicles Enriched in hsa-miR-301a-3p and hsa-miR-1293 Dynamics in Clear Cell Renal Cell Carcinoma Patients: Potential Biomarkers of Metastatic Disease
Source: Cancers (Basel). 2020 Jun 2;12(6):1450. doi: 10.3390/cancers12061450 (PMC7352268; doi:10.3390/cancers12061450)
Supplement: Supplementary file 1 [file cancers-12-01450-s001.pdf]

Supplementary Materials: Extracellular Vesicles Enriched in hsa-miR-301a-3p and hsa-miR-1293 Dynamics in Clear Cell Renal Cell Carcinoma Patients: Potential Biomarkers of Metastatic Disease

Table S1. Association of EV-derived miRNA profile expression with Clinical-pathological characteristics of patients with localized disease (Group A).

miRNA	Hypertension	Diabetes mellitus	Smokers vs Non-smokers	Smokers vs Ex-smokers	Tumor size > 7cm
Hsa-miR-25-3p	*P*=0.528	*P*=0.356	*P*=0.316	*P*=0.422	*P*=0.464
Hsa-miR-126-5p	*P*=0.528	*P*=0.814	*P*=0.108	*P*=0.079	***P*=0.013**
Hsa-miR-200c-3p	*P*=0.555	*P*=0.633	*P*=0.503	*P*=0.617	*P*=0.862
Hsa-miR-210-3p	*P*=0.382	*P*=0.996	*P*=0.211	***P*=0.034**	*P*=0.679
Hsa-miR-301a-3p	*P*=0.820	*P*=0.682	*P*=0.083	*P*=0.120	*P*=0.754
Hsa-miR-1246	*P*=0.958	*P*=0.056	*P*=0.770	*P*=0.558	*P*=0.572
Hsa-miR-1293	*P*=0.837	*P*=0.373	***P*=0.006**	*P*=0.577	*P*=0.194

Table S2. Hsa-miR-25-3p, hsa-miR-126-5p, hsa-miR-200c-3p and hsa-miR-301a-3p mRNA targets validated with strong evidence according miRTarBase V8.0.

microRNA	Validated targets
Hsa-miR-25-3p	*WDR4, TP53, TNFSF10, TCEAL1, SMAD7, SEMA4C, RGS3, REV3L, RECK, PTEN, PRMT5, MDM2, MAPK2K4, LATS2, KLF4, KAT2B, HAND2, FBXW7, EZH2, ERBB2, DSC2, DHFR, CYP2B6, CPEB1, CDKN1C, CDH1, CCL26, BCL2L11, ATP2A2*
Hsa-miR-126-5p	*SLC45A3, SPRED1, PTPN7, ADAM9, MMP7, CXCL12, MYC, VEGFA, HOTAIR, CRK, CYLD*
Hsa-miR-200c-3p	*ZNF217, ZFPM2, ZFPM1, ZEB2, ZEB1, XIAP, WDR37, VEGFA, VAC14, USP25, UBQLN1, TUBB3, TIMP2, TCF7L1, TBK1, SUZ12, SP1, SLC1A2, SIRT1, SHC1, SH3PXD2A, SEPT7, SEC23A, RPS6KB1, ROCK2, RNF2, RND3, RIND2, RHOA, RCOR3, RASSF2, PTPRD, PTPN13, PTEN, PRKCZ, PMAIP1, LEPR, KRAS, KLHL20, KLF9, KLF11, KDR, JAZF1, IKBKB, HOXB5, HFE, GEMIN2, GATA4, FOXO1, FN1, FLT1, FBLN5, ETS1, ERRFI1, ERBIN, ELMO2, EFNA1, EDNRA, E2F3, DUSP1, DNMT3B, DNMT3A, DNAJC3, DLC1, CRKL, CFL2, CDK2, CDH11, CCNE2, BTC, BRD7, BMI1, BCL2, BAP1, ATRX, ADAM12, ACVR2B*
Hsa-miR-301a-3p	*MEOX12, NKRF, SERPINE1, SMAD4, RUNX3, BCL2L11, PTEN, UVRAG, SNIP1, MAP3K5, TIMP2, CDC14A*

Table S3. Protein-protein-interaction (PPI) network clusters according to Marckov Clusterring (MCL) analysis.

Cluster	Clustered proteins
Cluster 1	*ZNF217, KLF9, RPS6KB1, KLF4, NOS3, TCF7L1, SUZ12, BRD7, E2F3, TCEAL1, RNF2, XIAP, SEC23A, ZEB1, ZEB2, KAT2B, SIRT1, ATRX, DNMT3A, DUSP1, DNMT3B, IKBKB, BMI1, PTEN, CDKN1C, EZH2, BAP1, PIN1, MDM2, BCL2, CDK2, SP1, CPEB1, DHFR, KLHL20, UVRAG, PMAIP1, BCL2L11, CYP2B6, FOXO1, PRKCZ, NOTCH1, DSC2, TP53, CCNE2, RUNX3, MAP3K5, TNFSF10, MYB, MYC, ETS1, SERPINE1*
Cluster 2	*DLC1, RHOA, RND3, MSN, CFL2, MYLK*
Cluster 3	*KRAS, SPRED1, PTPRD, SHC1, RASSF2*
Cluster 4	*VEGFA, SEMA4C, TIMP2, EFNA1, LEPR*
Cluster 5	*HAND2, ATP2A2, GATA4, ZFPM1, ZFPM2*
Cluster 6	*ERBB2IP, ERBB2, BTC, ERRFI1*
Cluster 7	*PTPN7, SEPT7, CDC14A*
Cluster 8	*ACVR2B, SMAD4, SNIP1*
Cluster 9	*PTPNI3, PDCD10, RGS3*
Cluster 10	*JAZF1, VAC14, SLC45A3*
Cluster 11	*NTF3, FLT1, KDR*
Cluster 12	*CRK, ELMO2, CRKL*
Cluster 13	*DNAJC3, MMP7, FN1*
Cluster 14	*CCL26, CXCL12*
Cluster 15	*RECK, SLC1A2*
Cluster 16	*USP25, WDR4*
Cluster 17	*NCAM1, TUBB3*
Cluster 18	*UBQLN1, TBK1*
Cluster 19	*CDH11, CDH1*
Cluster 20	*KLF11, SMAD7*
Cluster 21	*PRMT5, GEMIN2*
Cluster 22	*REV3L, ADAM9*
Cluster 23	*SH3PXD2A, ADAM12*
Cluster 24	*RCOR3, HFE*
Cluster 25	*FBLN5, RIN2*
Cluster 26	*LPAR1, EDNRA*

Table S4. Reactome pathway enrichment analysis for terms with FDR *p* value < 0.01.

Number of genes	Term name	Description	FDR value
23	HSA-74160	Gene expression (Transcription)	9,20E-11
11	HSA-2559583	Cellular Senescence	1,85E-10
20	HSA-212436	Generic Transcription Pathway	6,03E-10
27	HSA-162582	Signal Transduction	5,38E-09
8	HSA-8878159	Transcriptional regulation by RUNX3	1,51E-08
13	HSA-8953897	Cellular responses to external stimuli	1,51E-08
12	HSA-2262752	Cellular responses to stress	2,40E-08
7	HSA-2559580	Oxidative Stress Induced Senescence	3,72E-07
5	HSA-2559585	Oncogene Induced Senescence	2,17E-06
5	HSA-109606	Intrinsic Pathway for Apoptosis	6,61E-06
8	HSA-1257604	PIP3 activates AKT signaling	1,09E-05
6	HSA-212165	Epigenetic regulation of gene expression	2,51E-05
6	HSA-9018519	Estrogen-dependent gene expression	2,65E-05
4	HSA-114452	Activation of BH3-only proteins	4,21E-05
6	HSA-6807070	PTEN Regulation	4,68E-05
3	HSA-111453	BH3-only proteins associate with and inactivate anti-apoptotic BCL-2 members	6,23E-05
3	HSA-4411364	Binding of TCF/LEF:CTNNB1 to target gene promoters	6,23E-05
6	HSA-453279	Mitotic G1-G1/S phases	6,23E-05
3	HSA-8951430	RUNX3 regulates WNT signaling	6,23E-05
6	HSA-3108232	SUMO E3 ligases SUMOylate target proteins	7,61E-05
6	HSA-109581	Apoptosis	9.24E-5
8	HSA-3700989	Transcriptional Regulation by TP53	9.24E-5
4	HSA-212300	PRC2 methylates histones and DNA	9.8E-5
5	HSA-6785807	Interleukin-4 and Interleukin-13 signaling	1.6E-4
3	HSA-6804116	TP53 Regulates Transcription of Genes Involved in G1 Cell Cycle Arrest	1.6E-4
3	HSA-8941856	RUNX3 regulates NOTCH signaling	1.6E-4
3	HSA-4655427	SUMOylation of DNA methylation proteins	1.7E-4
4	HSA-8943724	Regulation of PTEN gene transcription	2.9E-4
4	HSA-1912408	Pre-NOTCH Transcription and Translation	3.4E-4
4	HSA-69563	p53-Dependent G1 DNA Damage Response	3.4E-4
4	HSA-170834	Signaling by TGF-beta Receptor Complex	5.0E-4
7	HSA-5663202	Diseases of signal transduction	5.4E-4
13	HSA-597592	Post-translational protein modification	5.5E-4
3	HSA-1538133	G0 and Early G1	6.5E-4
5	HSA-5633007	Regulation of TP53 Activity	7.1E-4
3	HSA-6804758	Regulation of TP53 Activity through Acetylation	8.3E-4
2	HSA-69200	Phosphorylation of proteins involved in G1/S transition by active Cyclin E:Cdk2 complexes	9.1E-4
3	HSA-2173796	SMAD2/SMAD3:SMAD4 heterotrimer regulates transcription	9.5E-4
6	HSA-5688426	Deubiquitination	9.5E-4
3	HSA-8953750	Transcriptional Regulation by E2F6	9.5E-4
4	HSA-6804756	Regulation of TP53 Activity through Phosphorylation	0.001

Table S5. KEGG pathway enrichment analysis. Only ther terms with FDR *P* value <0.01 were considered.

Number of genes	Term name	Description	FDR value
16	hsa05206	MicroRNAs in cancer	4,70E-19
18	hsa05200	Pathways in cancer	9,11E-14
11	hsa05215	Prostate cancer	2,43E-13
11	hsa04218	Cellular senescence	2,37E-11
9	hsa05222	Small cell lung cancer	1,80E-10
12	hsa04151	PI3K-Akt signaling pathway	3,82E-09
11	hsa05165	Human papillomavirus infection	1,95E-08
7	hsa04115	p53 signaling pathway	2,08E-08
8	hsa04068	FoxO signaling pathway	5,12E-08
8	hsa04210	Apoptosis	6,11E-08
7	hsa05210	Colorectal cancer	6,46E-08
8	hsa05161	Hepatitis B	7,43E-08
8	hsa05224	Breast cancer	8,88E-08
8	hsa05226	Gastric cancer	8,88E-08
7	hsa01522	Endocrine resistance	9,79E-08
6	hsa01524	Platinum drug resistance	4,77E-07
7	hsa04110	Cell cycle	4,77E-07
7	hsa05418	Fluid shear stress and atherosclerosis	7,33E-07
7	hsa05225	Hepatocellular carcinoma	2,61E-06
8	hsa05166	HTLV-I infection	2,96E-06
6	hsa04931	Insulin resistance	3.89E-6
6	hsa04919	Thyroid hormone signaling pathway	5.56E-6
6	hsa04071	Sphingolipid signaling pathway	5.58E-6
4	hsa04215	Apoptosis - multiple species	1.35E-5
5	hsa05220	Chronic myeloid leukemia	1.45E-5
4	hsa05219	Bladder cancer	3.48E-5
6	hsa05202	Transcriptional misregulation in cancer	3.86E-5
5	hsa04933	AGE-RAGE signaling pathway in diabetic complications	4.22E-5
6	hsa05203	Viral carcinogenesis	5.58E-5
6	hsa05169	Epstein-Barr virus infection	7.43E-5
4	hsa05213	Endometrial cancer	1.1E-4
4	hsa05221	Acute myeloid leukemia	1.7E-4
4	hsa05214	Glioma	1.8E-4
4	hsa05218	Melanoma	2.2E-4
4	hsa05212	Pancreatic cancer	2.3E-4
5	hsa04934	Cushing's syndrome	2.6E-4
4	hsa01521	EGFR tyrosine kinase inhibitor resistance	2.7E-4
4	hsa04211	Longevity regulating pathway	4.1E-4
4	hsa04066	HIF-1 signaling pathway	5.9E-4
3	hsa05216	Thyroid cancer	6.0E-4
4	hsa04722	Neurotrophin signaling pathway	0.001

Table S6. Gene Ontology (GO) Cellular Components enrichment analysis. Only the terms with FDR *p* value < 0.01 were considered.

Number of genes	Term name	Description	FDR value
37	GO.0005654	nucleoplasm	3,00E-14
39	GO.0031981	nuclear lumen	3,00E-14
40	GO.0070013	intracellular organelle lumen	9,87E-12
44	GO.0005634	nucleus	5,05E-11
52	GO.0043227	membrane-bounded organelle	2,68E-10
50	GO.0043231	intracellular membrane-bounded organelle	1,39E-09
47	GO.0044446	intracellular organelle part	1,55E-09
12	GO.0000790	nuclear chromatin	2,63E-09
52	GO.0043229	intracellular organelle	9,84E-09
15	GO.0044427	chromosomal part	6,21E-08
5	GO.0031519	PcG protein complex	7,95E-07
5	GO.0005720	nuclear heterochromatin	9,89E-07
31	GO.0032991	protein-containing complex	1,91E-06
31	GO.0005829	cytosol	4,14E-06
4	GO.0000803	sex chromosome	2,02E-05
4	GO.0000791	euchromatin	2,68E-05
11	GO.1990234	transferase complex	3,52E-05
11	GO.0016604	nuclear body	4,12E-05
13	GO.0044451	nucleoplasm part	4,17E-05
5	GO.0016605	PML body	7,59E-05
45	GO.0005737	cytoplasm	1.6E-4
7	GO.0005667	transcription factor complex	2.5E-4
2	GO.0097135	cyclin E2-CDK2 complex	3.5E-4
53	GO.0044464	cell part	4.2E-4
4	GO.1902911	protein kinase complex	7.0E-4

Table S7. Gene Ontology (GO) Biological data enrichment analysis. Only the terms with FDR *p* value < 0.01 were considered.

Number of genes	Term name	Description	FDR value
**31**	GO.0042127	regulation of cell population proliferation	5,21E-17
**48**	GO.0051171	regulation of nitrogen compound metabolic process	5,21E-17
**43**	GO.0031326	regulation of cellular biosynthetic process	7,62E-17
**39**	GO.0009893	positive regulation of metabolic process	7,95E-17
**48**	GO.0031323	regulation of cellular metabolic process	7,95E-17
**35**	GO.0031324	negative regulation of cellular metabolic process	7,95E-17
**38**	GO.0031325	positive regulation of cellular metabolic process	7,95E-17
**27**	GO.0051726	regulation of cell cycle	7,95E-17
**48**	GO.0060255	regulation of macromolecule metabolic process	7,95E-17
**48**	GO.0080090	regulation of primary metabolic process	7,95E-17
**42**	GO.2000112	regulation of cellular macromolecule biosynthetic process	7,95E-17
**34**	GO.0051172	negative regulation of nitrogen compound metabolic process	8,53E-17
**43**	GO.0048523	negative regulation of cellular process	8,56E-17
**37**	GO.0051173	positive regulation of nitrogen compound metabolic process	1,01E-16
**37**	GO.0010604	positive regulation of macromolecule metabolic process	4,36E-16
**20**	GO.0045786	negative regulation of cell cycle	5,86E-16
**41**	GO.0019219	regulation of nucleobase-containing compound metabolic process	6,42E-16
**34**	GO.0032268	regulation of cellular protein metabolic process	6,42E-16
**17**	GO.0035690	cellular response to drug	1,02E-15
**27**	GO.2000113	negative regulation of cellular macromolecule biosynthetic process	1,17E-15
**34**	GO.0010605	negative regulation of macromolecule metabolic process	1.19E-15
**42**	GO.0010468	regulation of gene expression	1.29E-15

**27**	GO.1901700	response to oxygen-containing compound	3.81E-15
**39**	GO.0051252	regulation of RNA metabolic process	5.82E-15
**38**	GO.0006355	regulation of transcription, DNA-templated	7.36E-15
**34**	GO.0010033	response to organic substance	1.55E-14
**33**	GO.0006357	regulation of transcription by RNA polymerase II	2.09E-14
**23**	GO.1901698	response to nitrogen compound	2.09E-14
**27**	GO.0033554	cellular response to stress	2.23E-14
**22**	GO.1901701	cellular response to oxygen-containing compound	3.77E-14
**22**	GO.0042493	response to drug	4.03E-14
**21**	GO.0000122	negative regulation of transcription by RNA polymerase II	7.01E-14
**27**	GO.0010941	regulation of cell death	7.5E-14
**28**	GO.0010628	positive regulation of gene expression	9.96E-14
**26**	GO.0042981	regulation of apoptotic process	9.98E-14
**27**	GO.0010629	negative regulation of gene expression	1.13E-13
**41**	GO.0048522	positive regulation of cellular process	1.41E-13
**25**	GO.0045934	negative regulation of nucleobase-containing compound metabolic process	3.09E-13
**23**	GO.0045892	negative regulation of transcription, DNA-templated	5.54E-13
**42**	GO.0048518	positive regulation of biological process	7.81E-13
**14**	GO.0010948	negative regulation of cell cycle process	1.25E-12
**11**	GO.0071236	cellular response to antibiotic	1.28E-12
**31**	GO.0070887	cellular response to chemical stimulus	1.94E-12
**12**	GO.0071453	cellular response to oxygen levels	1.94E-12
**26**	GO.0031399	regulation of protein modification process	2.73E-12
**44**	GO.0044260	cellular macromolecule metabolic process	3.7E-12
**23**	GO.0051253	negative regulation of RNA metabolic process	4.45E-12
**14**	GO.0046677	response to antibiotic	4.57E-12
**18**	GO.0008285	negative regulation of cell population proliferation	5.03E-12
**12**	GO.0000302	response to reactive oxygen species	8.51E-12
**14**	GO.0070482	response to oxygen levels	8.51E-12
**23**	GO.0009719	response to endogenous stimulus	8.95E-12
**16**	GO.0010035	response to inorganic substance	8.95E-12
**28**	GO.0050790	regulation of catalytic activity	1.21E-11
**36**	GO.0042221	response to chemical	1.83E-11
**19**	GO.0009725	response to hormone	2.14E-11
**19**	GO.0043066	negative regulation of apoptotic process	2.33E-11
**24**	GO.0051247	positive regulation of protein metabolic process	2.4E-11
**19**	GO.0010243	response to organonitrogen compound	3.14E-11
**10**	GO.0042542	response to hydrogen peroxide	3.78E-11
**13**	GO.0001666	response to hypoxia	3.95E-11
**12**	GO.0034599	cellular response to oxidative stress	4.31E-11
**32**	GO.0006950	response to stress	4.56E-11
**17**	GO.0010942	positive regulation of cell death	4.83E-11
**14**	GO.0006979	response to oxidative stress	4.9E-11
**23**	GO.0032270	positive regulation of cellular protein metabolic process	5.71E-11
**15**	GO.0009636	response to toxic substance	5.84E-11
**16**	GO.1901699	cellular response to nitrogen compound	6.06E-11
**17**	GO.0010564	regulation of cell cycle process	7.3E-11
**10**	GO.0034614	cellular response to reactive oxygen species	8.52E-11
**34**	GO.0048583	regulation of response to stimulus	1.04E-10
**27**	GO.0051128	regulation of cellular component organization	1.38E-10
**16**	GO.0043065	positive regulation of apoptotic process	1.4E-10
**21**	GO.0007049	cell cycle	1.63E-10
**51**	GO.0050794	regulation of cellular process	1.64E-10
**24**	GO.0045935	positive regulation of nucleobase-containing compound metabolic process	1.86E-10

**23**	GO.0051254	positive regulation of RNA metabolic process	1.86E-10
**22**	GO.0045893	positive regulation of transcription, DNA-templated	1.94E-10
**11**	GO.0097237	cellular response to toxic substance	2.15E-10
**10**	GO.0071456	cellular response to hypoxia	2.17E-10
**30**	GO.0009966	regulation of signal transduction	2.63E-10
**27**	GO.0050793	regulation of developmental process	3.67E-10
**26**	GO.0071310	cellular response to organic substance	3.81E-10
**31**	GO.0010646	regulation of cell communication	4.08E-10
**24**	GO.0031328	positive regulation of cellular biosynthetic process	4.09E-10
**8**	GO.0070301	cellular response to hydrogen peroxide	5.0E-10
**31**	GO.0023051	regulation of signaling	5.14E-10
**23**	GO.0045595	regulation of cell differentiation	5.42E-10
**46**	GO.0006807	nitrogen compound metabolic process	9.9E-10
**17**	GO.0033993	response to lipid	9.9E-10
**47**	GO.0044237	cellular metabolic process	9.9E-10
**23**	GO.0010557	positive regulation of macromolecule biosynthetic process	1.07E-9
**19**	GO.0071495	cellular response to endogenous stimulus	1.08E-9
**23**	GO.1902531	regulation of intracellular signal transduction	1.13E-9
**20**	GO.0051094	positive regulation of developmental process	1.6E-9
**40**	GO.0051716	cellular response to stimulus	2.2E-9
**13**	GO.1901652	response to peptide	3.21E-9
**23**	GO.2000026	regulation of multicellular organismal development	3.68E-9
**18**	GO.0009628	response to abiotic stimulus	4.14E-9
**47**	GO.0071704	organic substance metabolic process	4.36E-9
**46**	GO.0044238	primary metabolic process	7.81E-9
**27**	GO.0051239	regulation of multicellular organismal process	7.93E-9
**8**	GO.2000134	negative regulation of G1/S transition of mitotic cell cycle	9.01E-9
**17**	GO.0051338	regulation of transferase activity	9.38E-9
**14**	GO.0032870	cellular response to hormone stimulus	1.0E-8
**13**	GO.0071417	cellular response to organonitrogen compound	1.21E-8
**29**	GO.0065009	regulation of molecular function	1.24E-8
**12**	GO.2001233	regulation of apoptotic signaling pathway	1.27E-8
**20**	GO.0042325	regulation of phosphorylation	1.38E-8
**14**	GO.0007346	regulation of mitotic cell cycle	1.56E-8
**21**	GO.0019220	regulation of phosphate metabolic process	1.64E-8
**28**	GO.0006996	organelle organization	1.72E-8
**9**	GO.1902806	regulation of cell cycle G1/S phase transition	1.74E-8
**16**	GO.0014070	response to organic cyclic compound	1.84E-8
**17**	GO.0032269	negative regulation of cellular protein metabolic process	1.84E-8
**16**	GO.0008284	positive regulation of cell population proliferation	1.99E-8
**43**	GO.0050896	response to stimulus	2.39E-8
**35**	GO.0048856	anatomical structure development	2.57E-8
**28**	GO.0043412	macromolecule modification	2.63E-8
**10**	GO.0045930	negative regulation of mitotic cell cycle	2.65E-8
**19**	GO.0001932	regulation of protein phosphorylation	3.0E-8
**10**	GO.0048511	rhythmic process	3.4E-8
**14**	GO.0006325	chromatin organization	6.02E-8
**17**	GO.0045944	positive regulation of transcription by RNA polymerase II	6.02E-8
**12**	GO.0031667	response to nutrient levels	6.31E-8
**17**	GO.0044092	negative regulation of molecular function	7.21E-8
**33**	GO.0007275	multicellular organism development	8.94E-8
**18**	GO.0080134	regulation of response to stress	9.02E-8
**22**	GO.0048584	positive regulation of response to stimulus	1.08E-7
**16**	GO.0051276	chromosome organization	1.1E-7
**17**	GO.0033043	regulation of organelle organization	1.12E-7
**30**	GO.0090304	nucleic acid metabolic process	1.17E-7

**17**	GO.0009968	negative regulation of signal transduction	1.18E-7
**9**	GO.0043281	regulation of cysteine-type endopeptidase activity involved in apoptotic process	1.2E-7
**10**	GO.1901653	cellular response to peptide	1.2E-7
**9**	GO.0032868	response to insulin	1.37E-7
**15**	GO.0022402	cell cycle process	1.78E-7
**19**	GO.0051240	positive regulation of multicellular organismal process	1.99E-7
**24**	GO.0006351	transcription, DNA-templated	2.11E-7
**8**	GO.0031960	response to corticosteroid	2.11E-7
**15**	GO.0045597	positive regulation of cell differentiation	2.24E-7
**15**	GO.0051093	negative regulation of developmental process	2.29E-7
**6**	GO.2001244	positive regulation of intrinsic apoptotic signaling pathway	2.74E-7
**33**	GO.1901360	organic cyclic compound metabolic process	2.87E-7
**14**	GO.0043086	negative regulation of catalytic activity	4.11E-7
**19**	GO.0010647	positive regulation of cell communication	4.13E-7
**8**	GO.2001235	positive regulation of apoptotic signaling pathway	4.25E-7
**12**	GO.0010638	positive regulation of organelle organization	4.26E-7
**32**	GO.0006725	cellular aromatic compound metabolic process	4.33E-7
**19**	GO.0023056	positive regulation of signaling	4.33E-7
**18**	GO.0048585	negative regulation of response to stimulus	5.72E-7
**18**	GO.0009967	positive regulation of signal transduction	6.3E-7
**16**	GO.0031401	positive regulation of protein modification process	6.34E-7
**33**	GO.0016043	cellular component organization	7.5E-7
**25**	GO.0006464	cellular protein modification process	7.71E-7
**10**	GO.0043434	response to peptide hormone	7.87E-7
**12**	GO.0031400	negative regulation of protein modification process	8.52E-7
**27**	GO.0034645	cellular macromolecule biosynthetic process	8.73E-7
**27**	GO.0044271	cellular nitrogen compound biosynthetic process	9.23E-7
**15**	GO.0012501	programmed cell death	1.18E-6
**17**	GO.0043085	positive regulation of catalytic activity	1.19E-6
**7**	GO.1901796	regulation of signal transduction by p53 class mediator	1.19E-6
**13**	GO.0006974	cellular response to DNA damage stimulus	1.24E-6
**12**	GO.0080135	regulation of cellular response to stress	1.3E-6
**10**	GO.1901987	regulation of cell cycle phase transition	1.31E-6
**25**	GO.0018130	heterocycle biosynthetic process	1.36E-6
**25**	GO.0019438	aromatic compound biosynthetic process	1.39E-6
**27**	GO.0044267	cellular protein metabolic process	1.39E-6
**31**	GO.0046483	heterocycle metabolic process	1.46E-6
**8**	GO.0031669	cellular response to nutrient levels	1.49E-6
**10**	GO.0052548	regulation of endopeptidase activity	1.54E-6
**14**	GO.0006915	apoptotic process	1.58E-6
**16**	GO.0051336	regulation of hydrolase activity	1.58E-6
**7**	GO.0009267	cellular response to starvation	1.64E-6
**7**	GO.0051384	response to glucocorticoid	1.71E-6
**6**	GO.0043502	regulation of muscle adaptation	1.74E-6
**21**	GO.0051704	multi-organism process	1.76E-6
**6**	GO.1901216	positive regulation of neuron death	2.25E-6
**9**	GO.0050678	regulation of epithelial cell proliferation	2.38E-6
**32**	GO.0034641	cellular nitrogen compound metabolic process	2.42E-6
**14**	GO.1902533	positive regulation of intracellular signal transduction	2.64E-6
**6**	GO.0043535	regulation of blood vessel endothelial cell migration	2.66E-6
**7**	GO.0010212	response to ionizing radiation	2.68E-6
**10**	GO.0001933	negative regulation of protein phosphorylation	2.71E-6
**30**	GO.0044249	cellular biosynthetic process	2.74E-6
**6**	GO.0010822	positive regulation of mitochondrion organization	2.79E-6
**15**	GO.0051130	positive regulation of cellular component organization	2.81E-6

**25**	GO.1901362	organic cyclic compound biosynthetic process	2.81E-6
**53**	GO.0009987	cellular process	2.96E-6
**9**	GO.0001101	response to acid chemical	3.07E-6
**7**	GO.0032869	cellular response to insulin stimulus	3.08E-6
**9**	GO.0048545	response to steroid hormone	3.12E-6
**12**	GO.0045596	negative regulation of cell differentiation	3.22E-6
**7**	GO.2001236	regulation of extrinsic apoptotic signaling pathway	3.71E-6
**11**	GO.0045936	negative regulation of phosphate metabolic process	3.81E-6
**7**	GO.2001242	regulation of intrinsic apoptotic signaling pathway	3.84E-6
**18**	GO.0044093	positive regulation of molecular function	3.85E-6
**13**	GO.0060284	regulation of cell development	4.1E-6
**5**	GO.0043525	positive regulation of neuron apoptotic process	4.21E-6
**13**	GO.0043549	regulation of kinase activity	4.23E-6
**8**	GO.0071375	cellular response to peptide hormone stimulus	4.53E-6
**12**	GO.0043408	regulation of MAPK cascade	4.75E-6
**9**	GO.0045862	positive regulation of proteolysis	5.16E-6
**28**	GO.0048731	system development	5.17E-6
**9**	GO.1901990	regulation of mitotic cell cycle phase transition	5.59E-6
**9**	GO.0016569	covalent chromatin modification	6.39E-6
**13**	GO.0051270	regulation of cellular component movement	6.58E-6
**19**	GO.0009653	anatomical structure morphogenesis	6.74E-6
**4**	GO.0051570	regulation of histone H3-K9 methylation	6.79E-6
**5**	GO.0071385	cellular response to glucocorticoid stimulus	6.8E-6
**12**	GO.0030162	regulation of proteolysis	6.97E-6
**7**	GO.1904018	positive regulation of vasculature development	7.65E-6
**5**	GO.1902041	regulation of extrinsic apoptotic signaling pathway via death domain receptors	7.94E-6
**25**	GO.0016070	RNA metabolic process	7.97E-6
**11**	GO.0035239	tube morphogenesis	8.31E-6
**10**	GO.0071900	regulation of protein serine/threonine kinase activity	8.51E-6
**11**	GO.0051347	positive regulation of transferase activity	1.04E-5
**6**	GO.0045814	negative regulation of gene expression, epigenetic	1.1E-5
**18**	GO.0009605	response to external stimulus	1.13E-5
**12**	GO.0006366	transcription by RNA polymerase II	1.19E-5
**7**	GO.0048872	homeostasis of number of cells	1.2E-5
**12**	GO.0045859	regulation of protein kinase activity	1.25E-5
**13**	GO.0070647	protein modification by small protein conjugation or removal	1.26E-5
**12**	GO.0035295	tube development	1.32E-5
**8**	GO.1901214	regulation of neuron death	1.33E-5
**10**	GO.0044057	regulation of system process	1.35E-5
**6**	GO.0043280	positive regulation of cysteine-type endopeptidase activity involved in apoptotic process	1.41E-5
**29**	GO.1901576	organic substance biosynthetic process	1.43E-5
**25**	GO.0065008	regulation of biological quality	1.53E-5
**12**	GO.2000145	regulation of cell motility	1.55E-5
**6**	GO.0032355	response to estradiol	1.59E-5
**6**	GO.0048660	regulation of smooth muscle cell proliferation	1.59E-5
**6**	GO.0072331	signal transduction by p53 class mediator	1.72E-5
**9**	GO.1903706	regulation of hemopoiesis	1.83E-5
**7**	GO.0010632	regulation of epithelial cell migration	1.91E-5
**16**	GO.0035556	intracellular signal transduction	1.93E-5
**4**	GO.0090200	positive regulation of release of cytochrome c from mitochondria	1.94E-5
**6**	GO.0031056	regulation of histone modification	2.18E-5
**9**	GO.0009314	response to radiation	2.3E-5
**8**	GO.0033044	regulation of chromosome organization	2.3E-5
**6**	GO.0030856	regulation of epithelial cell differentiation	2.43E-5

**4**	GO.1903749	positive regulation of establishment of protein localization to mitochondrion	2.43E-5
**7**	GO.2001234	negative regulation of apoptotic signaling pathway	2.43E-5
**6**	GO.0009411	response to UV	2.61E-5
**9**	GO.0051346	negative regulation of hydrolase activity	2.84E-5
**7**	GO.0090257	regulation of muscle system process	2.84E-5
**5**	GO.0034644	cellular response to UV	2.98E-5
**25**	GO.0010467	gene expression	3.48E-5
**9**	GO.0030335	positive regulation of cell migration	3.6E-5
**6**	GO.0051147	regulation of muscle cell differentiation	3.6E-5
**6**	GO.0007050	cell cycle arrest	3.71E-5
**15**	GO.0048699	generation of neurons	3.79E-5
**4**	GO.0051443	positive regulation of ubiquitin-protein transferase activity	3.98E-5
**34**	GO.0032501	multicellular organismal process	4.2E-5
**8**	GO.0016570	histone modification	4.53E-5
**5**	GO.0031058	positive regulation of histone modification	4.53E-5
**20**	GO.0032879	regulation of localization	4.55E-5
**5**	GO.0097327	response to antineoplastic agent	4.72E-5
**11**	GO.0030334	regulation of cell migration	4.79E-5
**6**	GO.0043409	negative regulation of MAPK cascade	4.97E-5
**6**	GO.0097193	intrinsic apoptotic signaling pathway	4.97E-5
**6**	GO.2001252	positive regulation of chromosome organization	5.28E-5
**5**	GO.1901655	cellular response to ketone	5.43E-5
**7**	GO.0040029	regulation of gene expression, epigenetic	5.46E-5
**11**	GO.0048609	multicellular organismal reproductive process	5.5E-5
**6**	GO.0045766	positive regulation of angiogenesis	5.58E-5
**4**	GO.0070317	negative regulation of G0 to G1 transition	5.58E-5
**10**	GO.0003006	developmental process involved in reproduction	5.82E-5
**7**	GO.0007568	aging	5.9E-5
**9**	GO.0071396	cellular response to lipid	5.9E-5
**9**	GO.0001944	vasculature development	5.96E-5
**3**	GO.0014745	negative regulation of muscle adaptation	6.09E-5
**3**	GO.1902237	positive regulation of endoplasmic reticulum stress-induced intrinsic apoptotic signaling pathway	6.09E-5
**10**	GO.0000278	mitotic cell cycle	6.17E-5
**9**	GO.0010720	positive regulation of cell development	6.25E-5
**12**	GO.0022603	regulation of anatomical structure morphogenesis	7.5E-5
**9**	GO.0070848	response to growth factor	7.88E-5
**8**	GO.0051052	regulation of DNA metabolic process	7.99E-5
**9**	GO.1902532	negative regulation of intracellular signal transduction	8.7E-5
**4**	GO.0006306	DNA methylation	8.74E-5
**7**	GO.0070372	regulation of ERK1 and ERK2 cascade	8.75E-5
**14**	GO.0022414	reproductive process	9.18E-5
**7**	GO.0045765	regulation of angiogenesis	9.3E-5
**18**	GO.0007166	cell surface receptor signaling pathway	9.37E-5
**5**	GO.2001237	negative regulation of extrinsic apoptotic signaling pathway	9.57E-5
**18**	GO.0007399	nervous system development	9.79E-5
**21**	GO.0048513	animal organ development	9.81E-5
**6**	GO.1901654	response to ketone	9.86E-5
**11**	GO.0048646	anatomical structure formation involved in morphogenesis	1.1E-4
**14**	GO.0002682	regulation of immune system process	1.2E-4
**7**	GO.0009416	response to light stimulus	1.2E-4
**11**	GO.0018193	peptidyl-amino acid modification	1.2E-4
**8**	GO.0048608	reproductive structure development	1.2E-4
**5**	GO.0031398	positive regulation of protein ubiquitination	1.4E-4
**12**	GO.0034097	response to cytokine	1.4E-4

**6**	GO.0043523	regulation of neuron apoptotic process	1.4E-4
**3**	GO.0048070	regulation of developmental pigmentation	1.4E-4
**6**	GO.0008406	gonad development	1.5E-4
**23**	GO.0048869	cellular developmental process	1.5E-4
**15**	GO.0009888	tissue development	1.6E-4
**4**	GO.0010332	response to gamma radiation	1.6E-4
**4**	GO.0043536	positive regulation of blood vessel endothelial cell migration	1.6E-4
**8**	GO.0050769	positive regulation of neurogenesis	1.6E-4
**29**	GO.1901564	organonitrogen compound metabolic process	1.6E-4
**12**	GO.0045937	positive regulation of phosphate metabolic process	1.7E-4
**9**	GO.0016032	viral process	1.8E-4
**5**	GO.0000082	G1/S transition of mitotic cell cycle	1.9E-4
**5**	GO.0050680	negative regulation of epithelial cell proliferation	1.9E-4
**10**	GO.0050767	regulation of neurogenesis	1.9E-4
**4**	GO.0010660	regulation of muscle cell apoptotic process	2.1E-4
**4**	GO.0014910	regulation of smooth muscle cell migration	2.2E-4
**5**	GO.0016202	regulation of striated muscle tissue development	2.2E-4
**3**	GO.1902170	cellular response to reactive nitrogen species	2.2E-4
**9**	GO.0045664	regulation of neuron differentiation	2.4E-4
**12**	GO.0051241	negative regulation of multicellular organismal process	2.4E-4
**5**	GO.0071901	negative regulation of protein serine/threonine kinase activity	2.4E-4
**6**	GO.0090092	regulation of transmembrane receptor protein serine/threonine kinase signaling pathway	2.4E-4
**11**	GO.0044703	multi-organism reproductive process	2.5E-4
**8**	GO.0001568	blood vessel development	2.7E-4
**10**	GO.0019953	sexual reproduction	2.7E-4
**4**	GO.0070373	negative regulation of ERK1 and ERK2 cascade	2.7E-4
**8**	GO.1903827	regulation of cellular protein localization	2.8E-4
**4**	GO.0001776	leukocyte homeostasis	2.9E-4
**11**	GO.0001934	positive regulation of protein phosphorylation	2.9E-4
**4**	GO.0007569	cell aging	2.9E-4
**7**	GO.0048589	developmental growth	2.9E-4
**5**	GO.0007623	circadian rhythm	3.0E-4
**2**	GO.0014740	negative regulation of muscle hyperplasia	3.2E-4
**22**	GO.0030154	cell differentiation	3.3E-4
**9**	GO.0030155	regulation of cell adhesion	3.3E-4
**4**	GO.0045844	positive regulation of striated muscle tissue development	3.3E-4
**9**	GO.0007276	gamete generation	3.6E-4
**4**	GO.0008630	intrinsic apoptotic signaling pathway in response to DNA damage	3.6E-4
**4**	GO.0046902	regulation of mitochondrial membrane permeability	3.6E-4
**9**	GO.0051129	negative regulation of cellular component organization	3.6E-4
**3**	GO.0010661	positive regulation of muscle cell apoptotic process	3.8E-4
**9**	GO.0016567	protein ubiquitination	4.1E-4
**10**	GO.0072359	circulatory system development	4.1E-4
**3**	GO.0032461	positive regulation of protein oligomerization	4.2E-4
**3**	GO.1900740	positive regulation of protein insertion into mitochondrial membrane involved in apoptotic signaling pathway	4.2E-4
**6**	GO.0031647	regulation of protein stability	4.6E-4
**8**	GO.0071407	cellular response to organic cyclic compound	4.6E-4
**6**	GO.2000146	negative regulation of cell motility	4.8E-4
**9**	GO.0040008	regulation of growth	4.9E-4
**6**	GO.0090287	regulation of cellular response to growth factor stimulus	4.9E-4
**2**	GO.0072717	cellular response to actinomycin D	5.0E-4
**2**	GO.0090116	C-5 methylation of cytosine	5.0E-4
**2**	GO.1902263	apoptotic process involved in embryonic digit morphogenesis	5.0E-4
**26**	GO.0007165	signal transduction	5.4E-4

**3**	GO.0033032	regulation of myeloid cell apoptotic process	5.4E-4
**4**	GO.0048661	positive regulation of smooth muscle cell proliferation	5.4E-4
**3**	GO.0071549	cellular response to dexamethasone stimulus	5.4E-4
**6**	GO.1902105	regulation of leukocyte differentiation	5.5E-4
**6**	GO.0009410	response to xenobiotic stimulus	5.6E-4
**4**	GO.0030330	DNA damage response, signal transduction by p53 class mediator	5.8E-4
**3**	GO.0048730	epidermis morphogenesis	5.9E-4
**4**	GO.0097306	cellular response to alcohol	6.0E-4
**4**	GO.0048145	regulation of fibroblast proliferation	6.3E-4
**10**	GO.0009887	animal organ morphogenesis	6.7E-4
**27**	GO.0023052	signaling	6.7E-4
**3**	GO.0043500	muscle adaptation	7.0E-4
**3**	GO.0043516	regulation of DNA damage response, signal transduction by p53 class mediator	7.0E-4
**3**	GO.0045601	regulation of endothelial cell differentiation	7.0E-4
**2**	GO.0045636	positive regulation of melanocyte differentiation	7.0E-4
**2**	GO.0051097	negative regulation of helicase activity	7.0E-4
**5**	GO.2000377	regulation of reactive oxygen species metabolic process	7.0E-4
**4**	GO.0046620	regulation of organ growth	7.3E-4
**7**	GO.0051090	regulation of DNA-binding transcription factor activity	7.3E-4
**3**	GO.0003203	endocardial cushion morphogenesis	7.5E-4
**3**	GO.1904030	negative regulation of cyclin-dependent protein kinase activity	7.5E-4
**5**	GO.0018105	peptidyl-serine phosphorylation	7.6E-4
**6**	GO.0032102	negative regulation of response to external stimulus	7.7E-4
**4**	GO.0009791	post-embryonic development	7.8E-4
**6**	GO.0071214	cellular response to abiotic stimulus	7.9E-4
**3**	GO.0043029	T cell homeostasis	8.0E-4
**3**	GO.0048873	homeostasis of number of cells within a tissue	8.0E-4
**4**	GO.1904029	regulation of cyclin-dependent protein kinase activity	8.4E-4
**13**	GO.0042592	homeostatic process	8.7E-4
**4**	GO.0060070	canonical Wnt signaling pathway	8.7E-4
**27**	GO.0007154	cell communication	9.3E-4
**3**	GO.0034405	response to fluid shear stress	9.3E-4
**3**	GO.0110111	negative regulation of animal organ morphogenesis	9.3E-4
**2**	GO.1900378	positive regulation of secondary metabolite biosynthetic process	9.3E-4
**6**	GO.0030111	regulation of Wnt signaling pathway	9.4E-4
**4**	GO.0051153	regulation of striated muscle cell differentiation	9.6E-4
**3**	GO.0010907	positive regulation of glucose metabolic process	9.8E-4
**3**	GO.0014911	positive regulation of smooth muscle cell migration	9.8E-4
**3**	GO.0031062	positive regulation of histone methylation	9.8E-4
**3**	GO.1902042	negative regulation of extrinsic apoptotic signaling pathway via death domain receptors	9.8E-4
**6**	GO.0001525	angiogenesis	9.9E-4
**14**	GO.0051049	regulation of transport	9.9E-4
**3**	GO.0042149	cellular response to glucose starvation	0.001
**9**	GO.0051345	positive regulation of hydrolase activity	0.001
**5**	GO.0060485	mesenchyme development	0.001

Table S8. Gene Ontology (GO) Molecular Function data enrichment analysis. Only the terms with FDR *p* value < 0.01 were considered.

Number of genes	Term name	Description	FDR value
14	GO.0003682	chromatin binding	5,52E-09
42	GO.0005515	protein binding	5,52E-09
19	GO.0043565	sequence-specific DNA binding	5,52E-09
17	GO.0044212	transcription regulatory region DNA binding	5,52E-09
52	GO.0005488	binding	7,02E-09
15	GO.0000976	transcription regulatory region sequence-specific DNA binding	2,23E-08
15	GO.0019904	protein domain specific binding	2,23E-08
23	GO.0140110	transcription regulator activity	3,72E-08
14	GO.0000977	RNA polymerase II regulatory region sequence-specific DNA binding	4,83E-08
21	GO.0003700	DNA-binding transcription factor activity	5,19E-08
12	GO.0000987	proximal promoter sequence-specific DNA binding	1,14E-07
24	GO.0003677	DNA binding	1,14E-07
27	GO.0003676	nucleic acid binding	3,60E-07
22	GO.0019899	enzyme binding	3,72E-07
34	GO.1901363	heterocyclic compound binding	3,81E-07
19	GO.0000981	DNA-binding transcription factor activity, RNA polymerase II-specific	4,35E-07
34	GO.0097159	organic cyclic compound binding	5,09E-07
11	GO.0000978	RNA polymerase II proximal promoter sequence-specific DNA binding	6,26E-07
12	GO.0008134	transcription factor binding	1,15E-06
5	GO.1990841	promoter-specific chromatin binding	2,73E-06
11	GO.0019901	protein kinase binding	7.86E-6
9	GO.0001228	DNA-binding transcription activator activity, RNA polymerase II-specific	2.13E-5
4	GO.0051721	protein phosphatase 2A binding	2.3E-5
26	GO.0046872	metal ion binding	5.93E-5
18	GO.0140096	catalytic activity, acting on a protein	1.0E-4
5	GO.0042826	histone deacetylase binding	1.6E-4
5	GO.0019903	protein phosphatase binding	2.5E-4
4	GO.0002039	p53 binding	5.6E-4
2	GO.0003886	DNA (cytosine-5-)-methyltransferase activity	7.0E-4
3	GO.0035035	histone acetyltransferase binding	7.0E-4
2	GO.0050816	phosphothreonine residue binding	7.0E-4
4	GO.0008013	beta-catenin binding	7.7E-4
3	GO.0016538	cyclin-dependent protein serine/threonine kinase regulator activity	9.0E-4
30	GO.0043167	ion binding	9.0E-4
2	GO.0071535	RING-like zinc finger domain binding	0.0012
14	GO.0042802	identical protein binding	0.0014
16	GO.0016740	transferase activity	0.0016
10	GO.0044877	protein-containing complex binding	0.0019
2	GO.0001134	transcription regulator recruiting activity	0.0034
10	GO.0046914	transition metal ion binding	0.0034
4	GO.0002020	protease binding	0.0038
3	GO.0097110	scaffold protein binding	0.0039
2	GO.0004861	cyclin-dependent protein serine/threonine kinase inhibitor activity	0.0043
2	GO.0000900	translation repressor activity, mRNA regulatory element binding	0.0049
6	GO.0004674	protein serine/threonine kinase activity	0.0085
8	GO.0008270	zinc ion binding	0.0088
9	GO.0030234	enzyme regulator activity	0.0098
5	GO.0031625	ubiquitin protein ligase binding	0.0099
